# Encouraging People with Spinal Cord Injury to Take Part in Physical Activity in the COVID-19 Epidemic through the mHealth ParaSportAPP

**DOI:** 10.3390/healthcare10061069

**Published:** 2022-06-09

**Authors:** Adrià Marco-Ahulló, Lluïsa Montesinos-Magraner, Luís-Millan González, Teresa Crespo-Rivero, Patricia Launois-Obregón, Xavier García-Massó

**Affiliations:** 1 Departamento de Neuropsicobiología, Metodología y Psicología Social, Universidad Católica de Valencia, 46100 Valencia, Spain; adria.marco@ucv.es; 2 Spinal Cord Injury Unit, Physical Medicine and Rehabilitation, University Vall d’Hebron Campus, 08035 Barcelona, Spain; lluisa.montesinos@gmail.com (L.M.-M.); tcresporivero@gmail.com (T.C.-R.); 3 Department of Physical Education and Sport, FCAFE, Universitat de Valencia, 46010 Valencia, Spain; luis.m.gonzalez@uv.es; 4 Cardiorrespiratory Rehabilitation Unit, Physical Medicine and Rehabilitation, University Vall d’Hebron Campus, 08035 Barcelona, Spain; patricia.launois@gmail.com; 5 Departamento de Expresión Musical, Plástica y Corporal, University of Valencia, 46022 Valencia, Spain

**Keywords:** exercise, APP, spinal cord injury

## Abstract

Background: Although mHealth tools have great potential for health interventions, few experimental studies report on their use by people with spinal cord injuries in physical activity. Objective: The main objective of this study was to analyze the effect of the ParaSportAPP on different physical and psychological variables in people with paraplegia. Methods: Fourteen of these subjects made up the final sample. All the participants performed two pre-tests (control period) and a post-test with 8 months between the evaluations (COVID-19 broke out between pre-test 2 and the post-test). The ParaSportAPP was installed on their smartphones when they performed pre-test 2. The same tests were performed in the same order in all the evaluations: (i) the questionnaires PASIPD, HADS, RS-25; SCIM III and AQoL-8D, (ii) respiratory muscle strength, (iii) spirometry and (iv) cardiopulmonary exercise test. Results: The results showed no differences in any of the variables studied between the measurement times. Conclusions: Although none of the variables experienced improvements, the ParaSportAPP mobile application was able to lessen the impact of the pandemic on the variables studied.

## 1. Introduction

Suffering a spinal cord injury (SCI) is a dramatic event that affects physical and psychological health [1,2]. The severity of the SCI depends on the anatomical zone affected and the severity of the damage [3]. These injuries can lead to medical complications such as pressure ulcers, urinary tract infections, spasticity, musculoskeletal complications or respiratory and cardiovascular problems [1,4,5,6]. Maintaining adequate physical activity levels (PA) can be important in reducing and managing some of the secondary complications of the injuries listed above [7].

Although an active lifestyle can bring health benefits to this population, it seems that those with SCI carry out low levels of PA [8] due to different barriers and demotivating factors that make it difficult for them to get physical exercise (e.g., lack of knowledge about the exercises they can/should do, problems with the material or lack of economic resources to follow exercise programs), which usually leads to a reduced frequency of physical exercise [9]. This inactivity can lead to increased co-morbidity and poor physical fitness, which can severely affect this population’s quality of life and independence. Right now, the world is in the midst of an unprecedented situation due to the COVID-19 pandemic, which may further affect the PA levels of the SCI population [10].

Although many recent interventions have focused on promoting PA in those with SCI [11], currently, not all of them can be carried out with regularity because of COVID-19 restrictions (e.g., mobility constraints, closure or reduction of capacity of sports centers, etc.) [12,13]. Interventions based on tele-exercise platforms are thus of special interest. This sub-type of intervention can be relevant at this time, as the participants can carry out the procedure in their own homes.

These studies are performed through different devices such as computers or smartphones. Those conducted with smartphones can be a good option because they are possessed by a large part of the population and do not require an extra investment [14,15]. The features and functions of modern smartphones make them a valuable tool for transmitting and recording information on health-related variables. The specific apps aimed at managing health-related aspects (e.g., stopping smoking, calorie control or physical exercise) are known as mHealth apps and have been used in many interventions in populations with diabetes or hypertension [16]. However, few studies have focused on mHealth to promote PA in people with SCI [17]. 

Therefore, the present study aimed to analyze the effects of the mHealth ParaSportAPP, expressly created to promote PA in people with paraplegia, on quality of life, anxiety, depression, resilience, independence, and self-reported PA physical capacity of these people. In this way, it is expected that the use of this application will help improve the participants’ physical and psychological states.

## 2. Materials and Methods

### 2.1. Participants

Although 35 participants started the study, only 14 completed it. Of these, only 8 completed all the tests. The other 6 refused to take the PCR tests for fear of not being able to carry out the cardiorespiratory tests. The study experienced a substantial loss of the sample between pre-test 1 and pre-test 2 (13 participants), mostly for clinical reasons (detection of syringomyelia, surgical interventions, cardiac alterations during pre-test 1 evaluations) or due to the impossibility of contacting or travelling to the hospital. The 8 participants lost between pre-test 2 and the post-test were due to their fear of contracting COVID when travelling to the test center. The participants characteristics are shown in Table 1.

The inclusion criteria were: (i) an SCI between T2 and L5 with at least one year of evolution; (ii) being a full-time wheelchair user; (iii) having completely lost the motor function of their lower limbs, or a score of 0 on the lower limb items of the AIS scale; and (iv) having full-time use of a smartphone with the Android operating system.

The exclusion criteria were: (i) suffering from cognitive disorders and/or depression requiring intensive psychiatric treatment; (ii) post-traumatic cervical myelopathy; (iii) syringomyelia and/or motor or sensory impairment of the upper extremities; (iv) ischaemic cardiac disorders; (v) a recent osteoporotic fracture; (vi) being tracheotomized or ventilator-dependent; (vii) having an active neoplastic process, and/or (viii) ischial, sacral or trochanteric pressure ulcers.

All the subjects gave their informed consent for inclusion before they participated in the study, which was conducted in accordance with the Declaration of Helsinki. The study protocol was approved by the Hospital Universitari Vall d’Hebron’s Institutional Review Board (PR(ATR)85/2017 Identification Code), obtained on 28 January 2019.

### 2.2. Procedure

This study was based on an intrasubject repeated measures design in which only one group was required. All the subjects in this group completed three measurement sessions at 0, 8 and 16 months from the start of the study.

Sessions 1 and 2 (pre-test 1 and pre-test 2) were used as a control period. An intervention was carried out between sessions 2 and 3 (pre-test 2 and post-test) to promote PA through the ParaSportAPP mHealth app. Prior to the start of the intervention, the users were instructed in its use.

The same tests were carried out in the same order at all the testing times (pre-test 1, pre-test 2 and post-test). Participants performed the tests in the following sequence: (i) questionnaires: Physical Activity Scale for Individuals with Physical Disabilities (PASIPD), Hospital Anxiety and Depression Scale (HADS), Resilience Scale-25 (RS-25), Spinal Cord Independence Measure III (SCIM III) and Assessment of Quality of Life-8 Dimensions (AQoL-8D); (ii) respiratory muscle strength tests; (iii) spirometry and (iv) a cardiopulmonary exercise test. However, only 8 participants completed the respiratory muscle strength, spirometry and cardiopulmonary exercise tests in the post-test. The other 6 participants refused to take a PCR test (mandatory in the COVID epidemic for the respiratory tests).

### 2.3. Instruments

#### 2.3.1. Questionnaires

##### Self-Reported PA

The Spanish version of PASIPD was used to measure the participants’ self-reported PA. This scale was evaluated in Spanish-speaking people with an SCI in the study by Pérez-Tejero et al. [18].

Thirteen items on recreation, housework and occupational activities performed in the previous seven days composed this scale. Each item was answered, indicating the activities’ frequency and time of performance (days per week and time spent per day). The PASIPD final score was obtained by multiplying each item’s average hours per day by an item MET value (determined by the scale authors and reflecting the intensity of the activity performed).

##### Anxiety and Depression Levels

The information on anxiety and depression levels was registered through the Spanish version of the HADS [19]. This instrument is a self-reporting questionnaire consisting of 14 items. Two subscales can be distinguished within the same questionnaire that assesses anxiety (HADS-A) and depression (HADS-D), composed of 7 questions each.

##### Resilience

The Spanish version of the Resilience Scale-25 (RS-25) [20] was the questionnaire chosen to assess resilience in the subjects, as defined in relation to positive coping in the face of significant adversity [21].

##### Independence

The Spanish version of the Spinal Cord Independence Measure (SCIM-III) [22] was chosen to register the data relative to independence levels. This questionnaire, composed of 17 items, was designed specifically to measure levels of independence in people with an SCI, addressing all the relevant aspects of daily activities in this population.

##### Quality of Life

The Spanish translation of the Assessment of Quality of Life-8 Dimensions (AQoL-8D) was used to record the quality of life levels of the subjects [23]. AQoL-8D is a questionnaire of approximately 5 minutes’ duration consisting of 35 items used to assess 8 different dimensions of quality of life: (i) independent living; (ii) pain; (iii) senses; (iv) happiness; (v) coping; (vi) mental health; (vii) personal relationships and (viii) self-esteem.

#### 2.3.2. Respiratory Muscle Strength

A portable MicroRPM mouth-breathing manometer (CardinalHealth, Kent, UK) was used to measure inspiratory and expiratory muscle strength. This device was used in previous studies to measure maximal inspiratory pressure (PIM) and maximal expiratory pressure (PEM) in people with an SCI [24,25].

#### 2.3.3. Spirometry and Cardiopulmonary Exercise Test

An Ergoselect 400 arm cycle ergometer (Ergoline, Bitz, Germany) connected to the Vyntus CPX metabolic cart (Jaeger-Care-Fusion, Hoechberg, Germany) was used for effort testing.

The variables calculated for the cardiopulmonary exercise test were: oxygen consumption during the first ventilatory threshold (VO2VT1), oxygen consumption during the second ventilatory threshold (VO2VT2), maximal oxygen consumption (VO2 max), oxygen pulse, peak respiratory exchange ratio (RER) and peak power.

The cardiopulmonary exercise test was of incremental intensity, with 10 watts per minute load progression. The test began with 3 min of cardiorespiratory variables being recorded at rest, followed by 3 min of warm-up without a load. After this 6-min period, the test was started, and the load was increased to an intensity of 10 watts per minute. The exertion period ended when participants reported an inability to continue with the test (either for cardiorespiratory reasons or because they were unable to continue mobilizing the load at the specific pace). All the participants were instructed to maintain a steady pace of around 50 pedal strokes per minute (which they could see on the machine’s display). Once the exertion period was over, the participants remained at rest for 3 more minutes, after which the test was finished.

The metabolic cart was also used for spirometry, and the variables extracted from this test were forced vital capacity (FVC), forced expiratory volume in the first second (FEV1), and peak expiratory flow (PEF). Spirometry was performed with participants seated in wheelchairs and keeping their heads still. Following the recommendations of the American Thoracic Society and the European Respiratory Society, 3 valid spirometry tests were performed, of which the one with the highest values was recorded.

#### 2.3.4. ParaSportAPP

The ParaSportAPP is a novel mHealth app designed specifically to promote PA in people with paraplegia and full-time users of a manual wheelchair and expressly created for the present project. The ParaSportAPP has 79 physical exercises to work three physical capacities (strength, endurance and flexibility). The APP provides these physical exercises in two different ways: planned or on-demand. We refer to the provision of on-demand exercise when the user requests it, previously selecting the physical capacity he wants to work. The users can also choose to perform the exercise with or without equipment (dumbbells/elastic bands or water bottles/towels in the absence of equipment). Planned exercises were performed when the user agreed to carry out one of the five notifications that the application launches daily. These notifications contain a block of three randomly selected from the exercise database that does not require equipment (so they can perform the exercises if no equipment is available). The time when the exercise notification is delivered can be customized. An example of a planned activity suggested by the application can be the exercise “body weight lifting”, which, once presented to the user, will be accompanied by a GIF image of a model performing the task and the following message: “With your back straight and your hands resting on either side of the chair, raise your own weight upward, thus lifting your gluteus off the chair and back down again”.

This app can also register the PA performed in other activities (i.e., handbike, tennis, paddle tennis, etc.). Users simply click on the “Record” icon in the main menu and place the smartphone on the non-dominant arm using an armband or a suitable device holder to register these exercises. All PA is recorded on the smartphone using the device’s triaxial accelerometer. The number of minutes of PA performed at a moderate/vigorous intensity is calculated by the equation proposed in Marco-Ahulló et al. [26]. 

The ParaSportAPP is also able to provide daily feedback on the physical exercise performed. This information is transmitted to the user by means of a report that visually resembles a traffic light (red: far from achieving the target; yellow: close to achieving the target; green: target achieved). To discriminate between the 3 possible types of feedback, a classification system based on a decision tree is used. This decision tree uses as classification variables the number of exercises performed by the application and the number of minutes of physical exercise at moderate/vigorous-intensity recorded by the ParaSportAPP. Based on international recommendations [27], to achieve the daily target, the subjects should perform at least 12 of the exercises provided by the ParaSportAPP or 30 min of physical exercise at a moderate/vigorous intensity or an equivalent combination.

mHealth also includes 21 specific healthy lifestyle tips for the SCI population, which are provided through daily notifications (1 notification per day).

### 2.4. Data Analysis 

First, all the responses from the questionnaires were entered in an Excel document (Microsoft, Redmond, WA, USA) digitized, and the scores were calculated as described in the corresponding guidelines.

The values of each variable studied were calculated using the Vyntus CPX device for the cardiopulmonary exercise tests.

All the data were entered in the Statistical Package for Social Science Version 24 (SPSS Inc., Chicago, IL, USA, EEUU) for subsequent statistical analysis.

### 2.5. Statistical Analysis

First, the normality assumption was checked (*p* < 0.05) using the Kolmogorov–Smirnov test for all variables at each measurement time using the SPSS. If this assumption was met, parametric tests were applied; if not, non-parametric tests were applied.

In the case of the parametric tests, a repeated measures ANOVA (between the three measurement times) was first requested. If a significant effect was found, pairwise comparisons were performed using t-tests for related samples with Bonferroni correction.

For the non-parametric tests, Friedman’s ANOVA statistical test was applied first, and if an effect was found, Dunn’s test with Bonferroni correction was applied for pairwise comparisons. The significance level was set at *p* = 0.05 for all the analyses.

## 3. Results

### 3.1. Self-Reported PA

As a result of the statistical analysis, it was found that there were no differences in PASIPD total scores (χ^2^_2_ = 0.25; *p* = 0.88; ε^2^ = 0.019), recreational PA (χ^2^_2_ = 3.31; *p* = 0.19; ε^2^ = 0.25), housework PA (χ^2^_2_ = 4.158; *p* = 0.12; ε^2^ = 0.32) and occupational PA (χ^2^_2_ =2.0; *p* = 0.37; ε^2^ = 0.15) between measurement times (pre-test 1, pre-test 2 and post-test).

### 3.2. Anxiety, Depression and Resilience Levels

No intervention effect was found on the variables’ total score (χ^2^_2_ = 0.54; *p* = 0.76; ε^2^ = 0.041), the anxiety subscale (F_2,26_ = 0.096, *p* = 0.91, η^2^_p_ = 0.007), or the depression subscale (χ^2^_2_ = 0.2; *p* = 0.9; ε^2^ = 0.015) between measurement times. Likewise, no statistically significant differences were found between the scores of the RS-25 questionnaire according to measurement time (F_2,26_ = 3.16; *p* = 0.059; η^2^_p_ = 0.196).

### 3.3. Independence and Quality of Life

The statistical analysis did not show any effect for the intervention on the SCIM III questionnaire scores (F_1_._42,18_._42_ = 0.153; *p* = 0.785; η^2^_p_ = 0.012). In addition, no significant differences were observed in the quality of life variables of the total score (χ^2^_2_ = 0.04; *p* = 0.98; ε^2^ = 0.003), coping (χ^2^_2_ = 0.45; *p* = 0.79; ε^2^ = 0.035), pain (χ^2^_2_ = 2.54; *p* = 0.28; ε^2^ = 0.19), happiness (χ^2^_2_ = 3.95; *p* = 0.14; ε^2^ = 0.3), personal relationships (F_2,26_ = 0.41; *p* = 0.67; η^2^_p_ = 0.031), mental health (χ^2^_2_ = 2.21; *p* = 0.33; ε^2^ = 0.17), senses (χ^2^_2_ = 0.84; *p* = 0.66; ε^2^ = 0.064) and independent living (F_2,26_ = 0.68; *p* = 0.516; η^2^_p_ = 0.05) between measurement times, while there was an intervention effect on the self-esteem subscale (χ^2^_2_ = 6.0; *p* = 0.05; ε^2^ = 0.46), although no statistically significant differences were found between any of the measurement times after the pairwise comparison. The descriptive statistics for all the variables studied are shown in the Table 2.

### 3.4. Respiratory Muscle Strength, Spirometry and Cardiopulmonary Exercise Test

**Figure 1:**
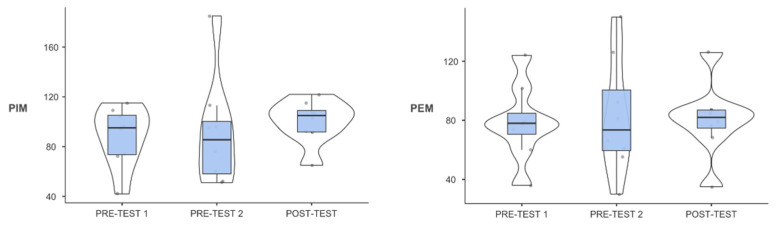
Box plots of PIM (**left**) and PEM (**right**) scores at the three measurement points. PIM = maximal inspiratory pressure; PEM = maximal expiratory pressure. *The dots show the position of the different subjects on the graph.

No differences were found between measurements in the variables PIM (F_2,14_ = 0.448; *p* = 0.648; η^2^_p_ = 0.06) and PEM (F_2,14_ = 0.082; *p* = 0.922; η^2^_p_ = 0.012) (Figure 1).

**Figure 2:**
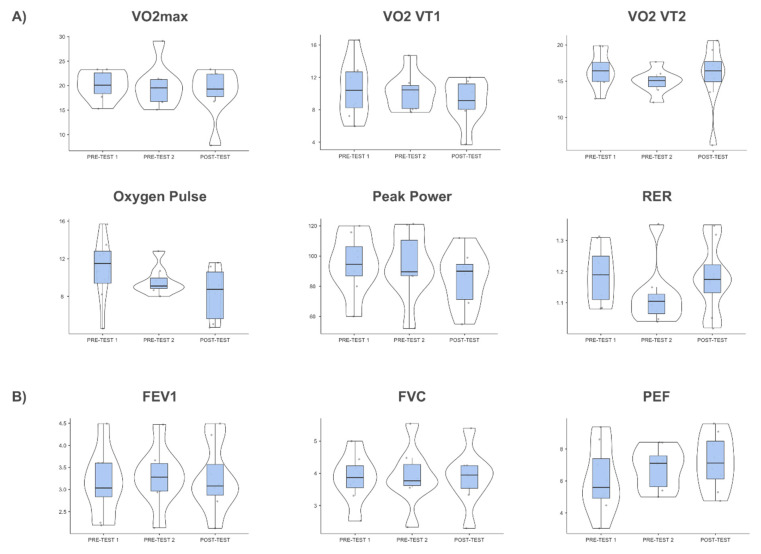
Box plots of cardiopulmonary exercise test (**A**) and spirometry (**B**) scores at the three measurement points. VO2max = maximal oxygen consumption; VO2 VT1 = oxygen consumption during first ventilatory threshold; VO2 VT2 = oxygen consumption during the second ventilatory threshold; RER = peak respiratory exchange ratio; FEV1 = forced expiratory volume in the first second; FVC = forced vital capacity; PEF = peak expiratory flow. *The dots show the position of the different subjects on the graph.

On the other hand, no differences were found between measurement times in the variables related to spirometry (FEV1 (F_2,14_ = 0.595; *p* = 0.565; η^2^_p_ = 0.078), FVC (F_2,14_ = 0.217; *p* = 0.81; η^2^_p_ = 0.03) and PEF (F_1_._34,9_._39_ = 3.613; *p* = 0 081; η^2^_p_ = 0.34)) and cardiopulmonary exercise testing (VO2VT1 (F_2,14_ = 1.231; *p* = 0.32; η^2^_p_ = 0.150), VO2VT2 (F_2,14_ = 0.646; *p* = 0.54; η^2^_p_ = 0.084), VO2 max (F_2,14_ = 0.824; *p* = 0.46; η^2^_p_ = 0.105), 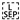
oxygen pulse (F_2,14_ = 2.578; *p* = 0.11; η^2^_p_ = 0.269), RER (F_2,14_ = 1.937; *p* = 0.18; η^2^_p_ = 0.217) and peak power (F_2,14_ = 2.48; *p* = 0.12; η^2^_p_ = 0.262)) (Figure 2).

## 4. Discussion

The main objective of this work was to carry out an intervention study through the novel ParaSportAPP mHealth app to promote PA and improve the physical and psychological variables of people with SCI confined to wheelchairs. For this, the following variables were monitored: quality of life, anxiety, depression, resilience, independence, self-reported PA and physical capacity (by means of effort and spirometry tests). As previously observed in the Results section, no differences were found between the three analyzed measurement times in any of the variables studied. It should also be pointed out that the COVID-19 pandemic broke out between the pre-test 2 and post-test measurements, which may have had a significant impact on these results.

If we focused on interventions for the promotion of PA in people with SCI, we can see some studies that evaluate variables such as quality of life and/or psychological variables [28,29,30,31,32]. In general terms, it seems that in the studies in which it was possible to increase PA levels and/or physical capacity, the participants’ quality of life also increased. However, this was not the case for psychological variables such as anxiety and depression.

If we focused on studies carried out by means of tele-exercise to evaluate psychological variables, we could highlight the one carried out by Chemtob et al. [28]. The authors of this paper conducted a pilot study in which they tried to promote PA through video conferencing with a PA specialist. The results showed that those in the experimental group showed higher autonomous motivation and leisure-time PA compared with the control group. Moderate to significant effects were also found in favor of the experimental group in terms of meaningful life experiences and social cognitive predictors of leisure-time PA, finding minor effects on variables such as depressive symptoms. It, therefore, seems that the intervention affected variables related to PA levels and quality of life but no changes in depressive symptoms.

mHealth is beginning to appear in the scientific literature as a tool for promoting health-related variables due to its scope and possibilities [33,34,35]. There are already some approaches to the application of these instruments for improving or controlling these variables in people with SCI [36,37,38,39]. The study by Canori et al. [38] is worth highlighting, focusing on the use of mHealth to promote PA in people with SCI. The authors used an mHealth app installed on a smartphone connected to a smartwatch, which recorded the PA performed by the users. The researchers used the feedback as a strategy to improve PA levels using real-time feedback and the JITAI methodology (providing information only in appropriate contexts and times). Although the authors did not apply statistical tests to compare the results obtained for each variable at each measurement time, by analyzing the descriptive statistics, it can be seen that PA levels were higher when using real-time feedback than in the control period or when feedback was provided by JITAI technology. In the case of the present study, feedback was provided when the user wanted to consult it. However, it did not give the number of minutes of PA performed, but the number of exercises carried out and the degree of achievement of the daily target. Additionally, it is essential to mention in this section the need to consider the accessibility of mHealth when focusing on people with disabilities. In the case of the application presented in this paper (ParaSportAPP), a study of its usability in the target population was conducted. The aforementioned study results classified the usability of the ParaSportAPP as “good” without finding significant differences between the values reported by the participants according to their level of injury [40].

Again, we should mention that COVID-19 may have partly conditioned the results obtained in this study. Recent research emphasizes the impact of the pandemic on the general population’s PA levels and psychological variables [41,42] and people with SCI specifically [10,43,44]. From this perspective, our results can be interpreted positively as the ParaSportAPP intervention may have had a mitigating effect on the impact of COVID-19 on the studied variables.

This study is not without its limitations. Its main weakness was the small sample size, conditioned by the dramatic mortality rate obtained due in part to COVID-19. The hospital where the work was carried out required that a negative PCR test accompany all the effort and spirometry tests performed no more than 48 h before the measurements (free of charge for the participants), which many participants refused to do. Finally, it is worth mentioning that the mHealth app was able to provide physical exercise according to the user’s capacity. The exercises were not chosen by the user but were provided randomly. This architecture was used so that the app would not be too large, which could lead to installation problems on the users’ devices. That said, it would be of great interest for future research to improve this aspect so that the participants can choose the exercises they want to consult.

In conclusion, this study used the novel ParaSportAPP mHealth app to promote PA and thus improve different physical and psychological variables in people with SCI. The results showed no differences between measurement points in any of the variables studied. These results, although apparently discouraging, can be interpreted as positive. It should be noted between pre-test 2 and the post-test, the COVID-19 pandemic broke out and finding no change could be interpreted as a lessening effect of the pandemic’s impact.

## Figures and Tables

**Table 1:** Participant characteristics.

	Age (Years)	Sex (Women/Men)	Thoracic Injury Level (High/Low)	Weight (kg)	Height (cm)	Injury Time (Years)
Only completed questionnaires (n = 14)	43.14 (9.49)	3/11	6/8	70.96 (12.92)	174.07 (9.37)	16.57 (11.03)
Completed all tests (n = 8)	47.87 (12.47)	1/7	3/5	71.94 (18.45)	173.25 (11.49)	20.5 (11.02)

Data are expressed as the mean (standard deviation).

**Table 2:** Descriptive statistics for each variable extracted from the questionnaires at each measurement point.

		Pre-Test 1	Pre-Test 2	Post-Test	*p*-Value	χ^2^_2_	F
Self-reported PA	PASIPD * Total	21.81 (20.4)	20 (14.37)	22.4 (29.69)	0.88	0.25	-
	PASIPD * Recreational activities	16.56 (15.16)	17.13 (9.34)	17.29 (17.65)	0.19	3.31	-
	PASIPD * Housework	1.39 (5.75)	1.17 (3.03)	2.01 (5.53)	0.12	4.16	-
	PASIPD * Occupational activities	0 (0)	0 (0)	0 (2.81)	0.37	2.00	-
Anxiety and Depression	HADS * Total	6 (4)	6.5 (4.25)	5.5 (6.25)	0.76	0.54	-
	HADS Anxiety	4 (2.83)	3.93 (2.81)	3.79 (2.91)	0.91	-	0.096
	HADS * Depression	2 (2.5)	2 (3.25)	2 (3.25)	0.90	0.20	-
Resilience	RS-25	151.43 (12.81)	150.42 (12.49)	145.29 (15.34)	0.06	-	3.16
Independence	SCIM III	68.88 (6.85)	68.57 (6.83)	68.43 (7.7)	0.78	-	0.153
Quality of Life	AQoL Total	80.15 (12,57)	81.2 (8.15)	80.85 (13.33)	0.98	0.04	-
	AQoL * Independent living	80.16 (11.67)	77.78 (13.25)	76.19 (15.77)	0.52	-	0.68
	AQoL Pain	70 (12.5)	80 (20)	75 (25)	0.28	2.54	-
	AQoL * Senses	96.15 (9.62)	92.31 (15.39)	96.15 (7.69)	0.66	0.84	-
	AQoL Happiness	81.25 (14.06)	78.12 (12.5)	81.25 (12.5)	0.14	3.95	-
	AQoL *Coping*	83.33 (16.66)	83.33 (16.66)	83.33 (16.66)	0.79	0.45	-
	AQoL * Mental health	80.3 (12.88)	78.78 (12.87)	78.78 (15.9)	0.33	2.21	-
	AQoL * Personal relationships	82.8 (7.63)	84.13 (7.47)	84.13 (9.57)	0.67	-	0.67
	AQoL *Self-esteem*	75 (16.66)	75 (16.66)	75 (18.74)	0.05	6.0	-

* All data are expressed as the mean (standard deviation), except for the variables marked with an asterisk, expressed as the median (interquartile range).

## Data Availability

The data presented in this study are available on request from the corresponding author.

## Nomenclature

AQoL8-D	Assessment of Quality of Life-8 Dimensions
FEV1	Forced Expiratory Volume in the first second
FVC	Forced Vital Capacity
HADS	Hospital Anxiety and Depression Scale
PA	Physical Activity
PASIPD	Physical Activity Scale for Individuals with Physical Disabilities
PEF	Peak Expiratory Flow
RER	Peak Respiratory Exchange Ratio
RS-25	Resilience Scale - 25
SCI	Spinal Cord Injury
SCIM III	Spinal Cord Independence Measure III
VO2max	Maximal oxygen consumption
VO2VT1	Oxygen Consumption during the First Ventilatory Threshold
VO2VT2	Oxygen Consumption during the Second Ventilatory Threshold
